# Reply to Open Letter

**Published:** 1890-08

**Authors:** W. Horace Hoskins

**Affiliations:** Secretary; Chairman of Sub-Committee of Arrangements


### CORRESPONDENCE.

### REPLY TO OPEN LETTER.

TO DR. W. L. WILLIAMS:

*Dear Sir:* In reply to the open letter published in the July number of the *Review*, permit me to make a few notes on the past history of the United States Veterinary Medical Association. It has always in the past confined its programme to members of its organization, and only by special request, given by a unanimous vote, allowed outside papers to be read at its meetings. I have personally attended these meetings since it honored me with an election, and I have yet to attend one which, to me, did not prove instructive, and I have yet to receive of one of its members a resignation on the grounds of failure to perform its duties. On my accession to the office of secretary a careful review of its work for the past twenty-five years, its records and lists of members, led me to feel that its greatest weakness was not in the ability and worth of its members, but in the one fact that the preponderance of its members was east of the Allegheny Mountains. Conferring with the higher officers of the association, and with their approval, I commenced, over one year ago, to develop an interest in the association among the Western members of the profession. In conjunction with our president, we carefully selected for resident state secretaries the best that our list afforded. With these members as a working body, I suggested to them the advisability of working up sufficient interest to command our association to again make the West a visit. After many months of persistent work, in the face of much opposition arising from reminiscences of the Cincinnati meeting, we brought sufficient influence to secure a meeting in Chicago for September, and now, for the first time, is sounded the alarm that the meeting is to be a failure. Whence comes the signal? From within the association's body? No; it comes from one outside of its number—from one who, but a few years since, had his name dropped for non-payment of initiation fee and dues. Should this, I ask, deter us from continuing the effort, lessening our zeal to exhibit to others the desire we have to fraternize with them, to mingle with them and make stronger the common ties that bind us together; make broader and grander the common work we are welded together to perform? And why are we thus criticised because we have adopted a plan to go to the West in a body, and, to make as good a showing as possible, have offered such inducements as we could secure to draw not only those who were favorable to a Western meeting of the association, but also those who were against the project? The manner in which we reach Chicago should be a matter of very little importance to any Westerner. We are not going there for a national show. We are visiting Chicago with only the interest of the whole profession at heart. We have chosen it for the place of our annual meeting for 1890 to prove unfounded the insinuation that we do not want Western veterinarians; to dispose of the argument that we do not join them because the distance to our meetings is too great, and that we never hold any accessible to the majority of our members. We have taken every method known to us to inform the entire West of the project, and have asked that the matter be brought before all your State associations, so that no one could again flaunt the excuse that the time and date of the meeting were unknown. We have lost no opportunity to avail ourselves of every offer made to make our programme instructive and highly entertaining, and no more lucid and valuable writers exist on this continent than are assured by the names of Profs. Liautard, Salmon and Huidekoper. No names of members from the West were debarred, nor have any requests from Western members of the association been denied. Our programme is not closed yet, and any new papers from any source will be announced. Surely, it was not expected that we, an association beyond the Allegheny Mountains, should seek the kindness and courtesies of the West, and prove so discourteous as to ask them not only to receive and entertain us, but also to provide the programme on which our minds and thoughts would feed. We may be lazy, but we are too gentlemanly to do this.

Our programme is not made by drafting members, but it is by solicitation on the part of the sub-committee, together with the officers. In this way we have secured the names of those already announced, and past experience of one-day meetings proved them too short for the transaction of our business and the proper hearing and discussion of one paper. It was for this reason more than any other that no further exertions have been made to enlarge the programme, as the long-continued sessions in the past, from 9 A. M. to 6, 7 or 8 P. M. have proved very tiresome, and thus adjournments without discussion or consideration of papers and reports of the utmost importance. This accounts largely to-day for the imperfect records of our association. To avoid this we had decided this year to utilize the first day to the transaction of routine business and hearing of committee reports, and in this direction have urged the various committees to make the most comprehensive reports possible, on the grounds of their being assured a full hearing and due consideration. This would leave the entire second day to the reading of papers and their discussion, and a knowledge of the subjects of three of these papers has lessened our zeal, in further extruding the efforts to enlarge the programme, because these papers will be fruitful of wide and interesting consideration and discussion. We yet await the announcement of the title of the fourth paper.

For the first time in the history of the association we have altered the law established by twenty-five years usage, that a name presented for membership should be laid over for consideration until the following meeting, thus affording proper time for the examination of the proposed member's credentials. This year, by special resolution of the *comitia minora*, we decided to hold open the list of applicants until September 1st, in order that the proposed members from the West would be enabled to become fully-fledged members in time for active participation in the transactions of the Chicago meeting. For exhibiting a disposition of unusul kindness to Western veterinarians, always in the past denied to applicants in the East, we are again severely criticised.

In answer to your questions:

1st. Our association's past record is the only answer we have to make in our dealings with graduates from regularly organized and recognized colleges; our list of members proves the worth of our record so far.

2d. The Chicago meeting is to be the twenty-seventh annual meeting of the United States Veterinary Medical Association.

3d. Our small list of members in the West, and their extreme modesty in not suggesting more than one of their number for a place on the programme, and our knowledge of the papers, importance so far offered for consideration at this meeting.

4th. The most cordial welcome it is within our power to extend to you; as to the unanimous election, I cannot vouch for that. It requires only a two-thirds vote for election, and this accomplished you will be accorded at this meeting the fullest privileges of membership that we can confer.

5th. No; we shall stand ready to make you further concessions as such may arise or be asked for, in the same spirit of kindness and fraternal bonds as those already conceded you.

We ask at your hands in Chicago, as has been conceded us in Boston, New York, Brooklyn, Philadelphia and Baltimore, a generous welcome, a strengthening of the fraternal relations and common purpose that draws us together, and a thorough amalgamation with us in all your strength of numbers, zeal of work and common interest in the noble structure given to our hands and minds to mould into a grand and completed work.

Very truly yours,

W. HORACE HOSKINS, *Secretary.*
*Chairman of Sub-Committee of Arrangements.*

